# Skull Vibration-Induced Nystagmus in Superior Semicircular Canal Dehiscence: A New Insight into Vestibular Exploration—A Review

**DOI:** 10.3390/audiolres14010009

**Published:** 2024-01-22

**Authors:** Georges Dumas, Ian Curthoys, Andrea Castellucci, Laurent Dumas, Laetitia Peultier-Celli, Enrico Armato, Pasquale Malara, Philippe Perrin, Sébastien Schmerber

**Affiliations:** 1 Department of Oto-Rhino-Laryngology Head and Neck Surgery, University Hospital, 38043 Grenoble, France; georges.dumas10@outlook.fr; 2 Research Unit 3450 DevAH—Development, Adaptation and Handicap, Faculty of Medicine, University of Lorraine, 54500 Vandoeuvre-lès-Nancy, France; laetitia.celli@gmail.com (L.P.-C.); enrico.armato@unipd.it (E.A.); philippe.perrin@univ-lorraine.fr (P.P.); 3 Vestibular Research Laboratory, School of Psychology, University of Sydney, Sydney, NSW 2006, Australia; ian.curthoys@sydney.edu.au; 4 ENT Unit, Department of Surgery, Azienda USL, IRCCS di Reggio Emilia, 42123 Reggio Emilia, Italy; andrea.castellucci@ausl.re.it; 5 INSERM UMR 1039 Bioclinic Radiopharmaceutics Laboratory, University Grenoble Alpes, 38700 La Tronche, France; laurent.dumas@univ-grenoble-alpes.fr; 6 Department of Neurosciences, University of Padova, 35100 Padova, Italy; 7 Audiology & Vestibology Service, Centromedico, 6500 Bellinzona, Switzerland; pasmalara@gmail.com; 8 Department of Paediatric Oto-Rhino-Laryngology, University Hospital of Nancy, 54500 Vandoeuvre-lès-Nancy, France; 9 INSERM UMR 2015, Brain Tech Laboratory, 38700 La Tronche, France

**Keywords:** vertigo, Minor syndrome, Tullio, Hennebert sign, skull-vibration-induced nystagmus

## Abstract

The third window syndrome, often associated with the Tullio phenomenon, is currently most often observed in patients with a superior semicircular-canal dehiscence (SCD) but is not specific to this pathology. Clinical and vestibular tests suggestive of this pathology are not always concomitantly observed and have been recently complemented by the skull-vibration-induced nystagmus test, which constitutes a bone-conducted Tullio phenomenon (BCTP). The aim of this work was to collect from the literature the insights given by this bedside test performed with bone-conducted stimulations in SCD. The PRISMA guidelines were used, and 10 publications were included and analyzed. Skull vibration-induced nystagmus (SVIN), as observed in 55 to 100% of SCD patients, usually signals SCD with greater sensitivity than the air-conducted Tullio phenomenon (ACTP) or the Hennebert sign. The SVIN direction when the test is performed on the vertex location at 100 Hz is most often ipsilaterally beating in 82% of cases for the horizontal and torsional components and down-beating for the vertical component. Vertex stimulations are more efficient than mastoid stimulations at 100 Hz but are equivalent at higher frequencies. SVIN efficiency may depend on stimulus location, order, and duration. In SCD, SVIN frequency sensitivity is extended toward high frequencies, with around 400 Hz being optimal. SVIN direction may depend in 25% on stimulus frequency and in 50% on stimulus location. Mastoid stimulations show frequently diverging results following the side of stimulation. An after-nystagmus observed in 25% of cases can be interpreted in light of recent physiological data showing two modes of activation: (1) cycle-by-cycle phase-locked activation of action potentials in SCC afferents with irregular resting discharge; (2) cupula deflection by fluid streaming caused by the travelling waves of fluid displacement initiated by sound or vibration at the point of the dehiscence. The SVIN direction and intensity may result from these two mechanisms’ competition. This instability explains the SVIN variability following stimulus location and frequency observed in some patients but also discrepancies between investigators. SVIN is a recent useful insight among other bedside examination tests for the diagnosis of SCD in clinical practice.

## 1. Introduction

Patients with superior semicircular canal dehiscence (SCD) often have atypical presentations but usually show audiovestibular symptoms consistent with mild conductive hearing loss associated with an unusual “too good” bone conduction (negative bone conduction thresholds for low frequencies on pure tone audiometry), tinnitus, hyperacusis, autophony, and often atypical vestibular symptoms such as positional vertigo, vertigo induced by noise, pressure-induced vertigo, or Menière-like attacks of vertigo [1,2,3,4]. There is sensitivity to external or middle-ear pressure variations (Hennebert sign, Valsalva test). Eye movements and sway or imbalance induced by low-frequency air-conducted (AC) sounds at high intensity have been described by Tullio in animals [5,6] and by Parker (250–2000 Hz at 100–120 dB intensity) in humans [7]. Similar eye movements or nystagmus associated with dizziness have been reported after cranial bone vibrations applied with the skull-vibration-induced nystagmus test (SVINT) [8,9,10].

Vestibular-evoked myogenic potentials (VEMPs), including ocular VEMPs (oVEMPs) and cervical VEMPs (cVEMPs), are considered as the gold-standard tests in SCD, showing a hypersensitivity of the utricle and sacculus, with large amplitudes and lower thresholds in response to air- or bone-conducted sound stimulations [11,12,13,14,15]. Unlike the responses of healthy subjects, there are responses to very-high-frequency stimulation (4000 Hz) in this pathology [13].

Nystagmus induced by vibrations of the cranium (VIN) in patients exhibiting sound-induced vertigo was reported for the first time at the XX^th^ Barany Society in 1998 [16] and first recorded in 2005 [8]. Since then, many authors [9,10,17,18,19,20,21,22,23] have published different series about bone-conducted (BC) skull-vibration-induced nystagmus (SVIN) or ocular movements (vibration-induced vestibulo-ocular reflex (ViVOR)) in patients with SCD. Some results in these publications differ because of different protocols and different vibration locations or frequencies used for the stimulus [24]. This review seeks to explain the likely cause of some variable results based on physiological evidence.

The SVIN test is now considered as a bone-conducted Tullio phenomenon (BCTP) and is part of the armamentarium of bedside examination tests for SCD diagnosis [24].

Our systematic review quantified the main characteristics, sensitivity, and utility of SVIN in SCD from a clinician’s and physician’s holistic perspective.

## 2. Methodology

This study was conducted in accordance with the Preferred Reporting Items for Systematic Reviews and Meta-analysis (PRISMA) checklist and recommendations.

### 2.1. Search Strategy

Articles indexed in PubMed/Medline and Scopus were searched for the last two decades from 2000 to June 2023, and selection was performed according to the PRISMA guidelines.

These search criteria were “Vibration induced Nystagmus” [all fields], cranial vibrations [all fields], and superior canal dehiscence [all fields]. Two independent investigators (GD and PP) reviewed the articles extracted from the literature review. Duplicates were removed, and each reviewer singularly filled in an Excel data sheet (Microsoft Corporation, USA) including information extracted from the articles. Files were then compared, and disagreements on the inclusion/exclusion papers were debated until complete agreement between researchers was achieved. Only papers that received full consensus were considered. The PRISMA guidelines were followed to conduct the systematic review.

### 2.2. Study Selection Criteria

Only publications concerning patients who corresponded to the superior SCD definition given by Minor et al. [1], with the characteristics summarized by Ward et al. [4], were included in this review paper.

### 2.3. Study Data Extraction

Sixteen publications were initially included in the flowchart, but six were not retained because of a small series of patients (below five) and/or because of insufficient inclusion criteria.

**Figure 1:**
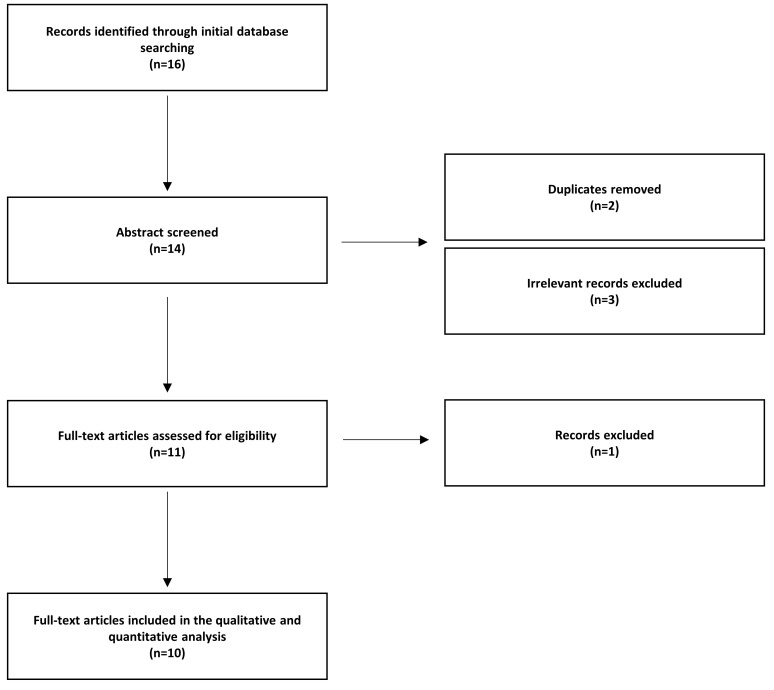
Flow chart illustrating how research into articles was performed following the PRISMA guidelines. The search criteria were “Vibration induced Nystagmus” [all fields], cranial vibrations [all fields], superior canal dehiscence [all fields]. Two independent investigators reviewed the articles extracted from the literature review.

The flow chart is presented in Figure 1. A chronological presentation of these papers is detailed below, and a summary is given in Table 1.

## 3. SVIN Results in the SCD

White et al. (2007) [10] analyzed at 100 Hz stimulation a series of eight patients with SCD using a 2D video-nystagmograph (VNG) recording the horizontal and vertical components and observed the torsional component under videoscopy. Patients stimulated on mastoids, vertex, or suboccipital region showed vibration-induced nystagmus (VIN) with torsional (most often ipsilaterally beating in three of five cases) and/or vertical components (most often down-beating in three of four cases with a vertical component). These authors suggested that the responses were due to stimulation of the superior dehiscent semicircular canal (SCC).

Schmerber et al. (2008) [22] studied a series of six patients with SCD stimulated at 100 Hz on mastoids and vertex and recorded using a VNG in 2D. These authors reported an SVIN with a horizontal component ipsilaterally beating toward the affected side, as identified by the ear with the air–bone gap in three cases and a vertical component in four cases.

Manzari et al. (2008) [19] analyzed the SVIN with a 3D device in a series of 16 SCD patients stimulated at 100 Hz on mastoids or vertex and observed in all patients a consistent torsional component, most often ipsilaterally beating toward the lesion (nystagmus in eight of nine uSCD patients [88%]). In bilateral SCD (bSCD), the nystagmus was stronger, ipsilaterally beating toward the predominant symptomatic side. The SVIN horizontal component was small and often (in 62% of cases) the direction changed following stimulation of the mastoid side. The vertical component was most often down-beating, but the change in direction was dependent on the side of the mastoid stimulated in 60% of cases. Conversely, an AC Tullio phenomenon (ACTP) was less frequently observed in 9/16 cases (56%).

Aw et al. (2011) [18] showed in a series of 17 SCDs recorded with scleral coils (3D analysis) that the slow phase of the vibratory vestibulo-ocular reflex (ViVOR) identified vertical semicircular canal dehiscence (SCD) and suggested that the vertical component was higher in patients with bilateral SCD (bSCD) than in those with unilateral SCD (uSCD). These patients fixed a target at 600 mm and were stimulated with short bone-conducted vibration (BCV) stimuli at 500 Hz. The ViVOR horizontal component was described as very small or negligible.

Dumas et al. (2014) [17] reported that in 17 unilateral SCD (uSCD) patients, stimulated with BCV at 100 Hz for 10 s without visual fixation, SVIN was observed in 82% of cases. Torsional, mainly vertical (most often up-beating in 80%), and horizontal components were observed in 30%, 30%, and 40% of cases, respectively. In these patients, vertex (Vx) stimulation was very efficient to provoke a nystagmus, and the SVIN torsional and horizontal components were most often beating ipsilaterally to the lesion (in 100% and in 80% of positive cases after Vx and mastoid stimulations, respectively) in opposition to what is observed in uVL patients who usually present SVIN beating away from the lesion side. Thus, it was suggested by analogy to consider SVIN as a vestibular Weber test. Some discrepancies following the stimulus location in the SVIN direction obtained after right (RM) or left mastoid (LM) stimulations were observed. Hence, vertex stimulation was recommended as a referential.

**Figure 2:**
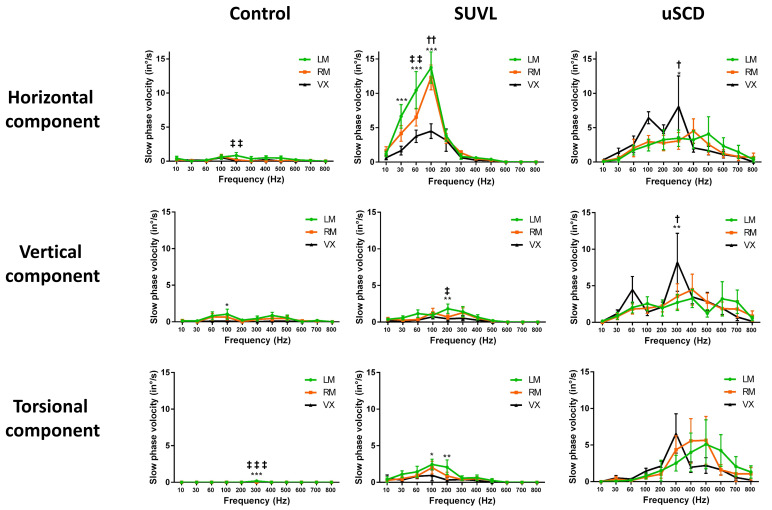
SVIN characteristics in uSCD vs. controls and uVL patients (optimal frequencies—optimal locations—SVIN components). Three-dimensional recordings of the SVIN slow-phase velocity (modified from Dumas et al., 2019 [9]). The stimulator (mini shaker; Bruel & Kjaer; Naerum; Denmark) delivered frequencies from 10 to 800 Hz. In controls (n = 11): no or no significant responses (inconsistent SVIN direction; slow-phase velocity < 2.5°/s). In unilateral superior canal dehiscence (uSCD) (n = 14): higher responses are observed on vertex and mastoids around 400–500 Hz but are not significantly higher than at 100 Hz (tendency). The sensitivity is extended to a wide range of frequencies (60 to 800 Hz) to elicit responses. The vertex location shows higher responses than the mastoid location at 100 and 300 Hz but not for other frequencies. On mastoids at 500 Hz, the SVIN-SPV is 4.10 ± 2.51°/s. The 3 components (H, V, T) are equally represented. In severe unilateral vestibular lesions (SUVLs) (n = 18) (lesions in patients with encased labyrinth): responses are significantly higher at 100 Hz (*p* < 0.001) on mastoid location (SVIN-SPV mean values: on left mastoid LM: 13.75 ± 2.28°/s and on right mastoid RM: 12.33 ± 1.81°/s). No responses are observed beyond 300 Hz. The horizontal component is mainly represented, with poor vertical responses. LM = left mastoid; RM = right mastoid; Vx = vertex. SPV = slow-phase velocity of the nystagmus. *; **; *** indicates significance at *p* < 0.05, <0.01, and <0.001 respectively, when comparing LM/Vx. †; ††; indicates significance at *p* < 0.05, and <0.01 respectively, when comparing RM/Vx. ‡; ‡‡; ‡‡‡ indicates significance at *p* < 0.05, <0.01, and <0.001 respectively, when comparing LM/RM.

**Figure 3:**
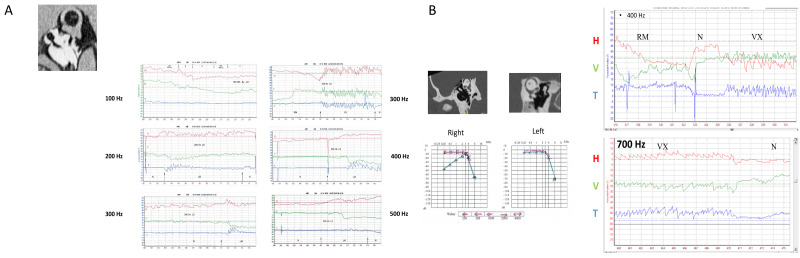
Extension of frequency sensitivity toward very high frequencies (modified from Dumas et al., 2019 [9]). (**A**) Example of a right SCD with Meniere-like attacks of vertigo. Responses are recorded up to 500 Hz. Poor responses are recorded at 100 Hz. Greater responses are observed at 300 and 400 Hz. The directions of horizontal, vertical, and torsional components change with the frequency (100 Hz vs. 300 and 500 Hz). (**B**) Example of a right uSCD recorded at 400 and 700 Hz. This patient had very poor responses at 100 Hz.

In another series of 40 patients with SCD (27 uSCD–13 bSCD) analyzed with 2D or 3D VNG recordings, Dumas et al. (2019) [9] reported a significantly higher efficiency of vertex *versus* mastoid stimulations at 100 and 300 Hz (but not at higher frequencies) to elicit an SVIN. Vertex location efficiency was higher in uSCD than in unilateral peripheral vestibular loss (uVL) patients. The results of vertex stimulation showed a response in more than 80% of uSCD. The torsional and horizontal components showed a nystagmus beating ipsilaterally to the lesion in 95% of positive cases. An equal representation of horizontal, vertical, and torsional components was observed in uSCD (Figure 2). These authors suggested that the superior SCC was not the only stimulated structure (it explained only the torsional and vertical component) and was associated with a likely concomitant utricle stimulation (or lateral SCC) contribution explaining the horizontal component. The optimal frequency was around 400 Hz and confirmed a sensitivity extended toward very high frequencies up to 500–700 Hz (Figure 3) when compared with sensitivity in UVL limited to frequencies around 100 Hz (in uVL patients, no responses were observed at 500 Hz) (Figure 2 and Figure 3). An SVIN horizontal component was more often observed in patients with uSCD (85%) than in those with bSCD (23%). In this series, the vertical component was up-beating in 66% of the cases. The slow-phase velocity (SPV) of horizontal and torsional components tended to be lower in bSCD than in uSCD. A vertical component was as frequently observed in bSCD as in uSCD in patients stimulated on mastoids. The SVIN torsional or horizontal component direction on each mastoid was not always concordant, and vertex stimulation was considered as referential to indicate the side of the nystagmus quick-phase. Conversely, in uVL patients, the SVIN direction was described as usually invariable regardless of mastoid or vertex stimulation location and beating toward the intact side.

The observation of a horizontal component corresponding to the slow phase of the vibratory vestibulo-ocular reflex (ViVOR) in SCD was confirmed by Park et al. (2014) [20] in 60% of their 10 patients when ocular fixation was denied. These authors attributed it to the horizontal SCC co-stimulation and underlined that skull vibrations induce an excitatory nystagmus for the horizontal components and that the nystagmus vertical component is weak and most often down-beating.

Mehta et al. (2015) [23] observed, in a series of 38 definite SCD patients, a positive Hennebert sign in 21%, a positive Tullio phenomenon in 26%, and a positive SVIN in 55% [24]. The stimulator mini massager (Merrimack, NH, USA) was applied only to the mastoid processes.

Batuecas et al. (2022) [21] reported that in a series of 30 patients with SCD stimulated at 100 Hz (VVIB, Synapsys, Marseille, France) on the mastoid and vertex and recorded by a 2D VNG, SVIN was positive in 66% of cases with a dominant horizontal component in 20% and a dominant vertical up-beating nystagmus in 46% of cases.

Dumas et al. recently reported in 2023 [24] data previously presented at the Barany Society Meeting in Madrid in 2022, which confirm that SVIN acts as a BC Tullio phenomenon. These last studies sought to link SVIN records to recent physiology and are reported in Table 1 and Table 2.

These authors included 52 patients (39 uSCD and 13 bSCD) according to the SCD criteria described by Ward et al. [4]. The patients were studied with the same protocol except for the order of stimulation location: in 12 uSCD patients, the LM and RM were first stimulated and then the vertex (GD order of stimulation protocol); 27 other uSCD patients were first stimulated on the Vx location and then on mastoids (AC order of stimulation protocol) (Table 1 and Table 2). Recordings were performed either with VNG (2D–3D) or video movies.

The sensitivities of SVIN in the 39 uSCD either observed after mastoid and/or vertex location stimulations were 91% and 93% of cases in these protocols, respectively (Table 2).

**Figure 4:**
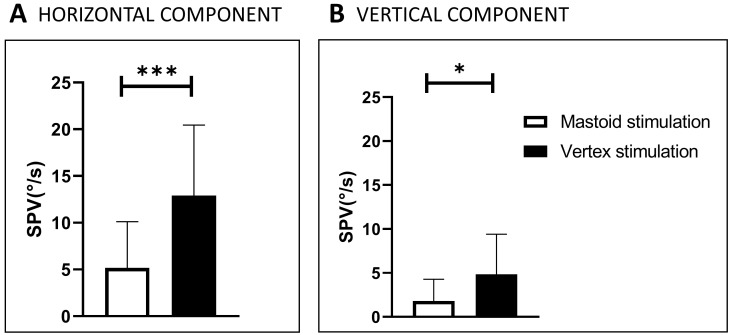
SVIN-SPV in 21 SCD patients stimulated on mastoids and vertex at 100 Hz (modified from Dumas et al., 2019 [9]). The order of stimulation was as follows: left mastoid (LM) for 10 s, right mastoid (RM) (for 10 s), and then vertex (Vx) for 10 s. SPV: slow-phase velocity of the nystagmus horizontal component (**A**) and of the vertical component (**B**). SVIN: skull-vibration-induced nystagmus. Significant difference: *** = *p* < 0.001. Significant difference: * = *p* < 0.05. The black plots represent vertex stimulation results, the white plots correspond to mastoid stimulation results. For the Horizontal component (**A**): Vertex stimulation SVIN-SPV: 12.9°/s ± 7.5. Mastoid SVIN SPV: 5.2 ± 4.9°/s (*p* < 0.005). For the vertical component (**B**): Vertex SVIN-SPV: 4.8 ± 4.6, Mastoid: 1.8 ± 2.5°/s, (*p* < 0.05).

In the first order of stimulation, the most efficient location was Vx (SVIN observed in 75%), and Vx stimulation was as efficient as mastoid stimulation in 25% of the other patients. The horizontal component SVIN-SPV at vertex locations was 12.9°/s ± 7.5, and it was 5.2 ± 4.9°/s at mastoid locations (*p* < 0.005), as reported in a previous work [9] (Figure 4). In the second order of stimulations, mastoid stimulation was more efficient in 58% of cases, Vx in 8%, and Vx stimulation was as efficient as mastoid stimulation in 35%.

**Figure 5:**
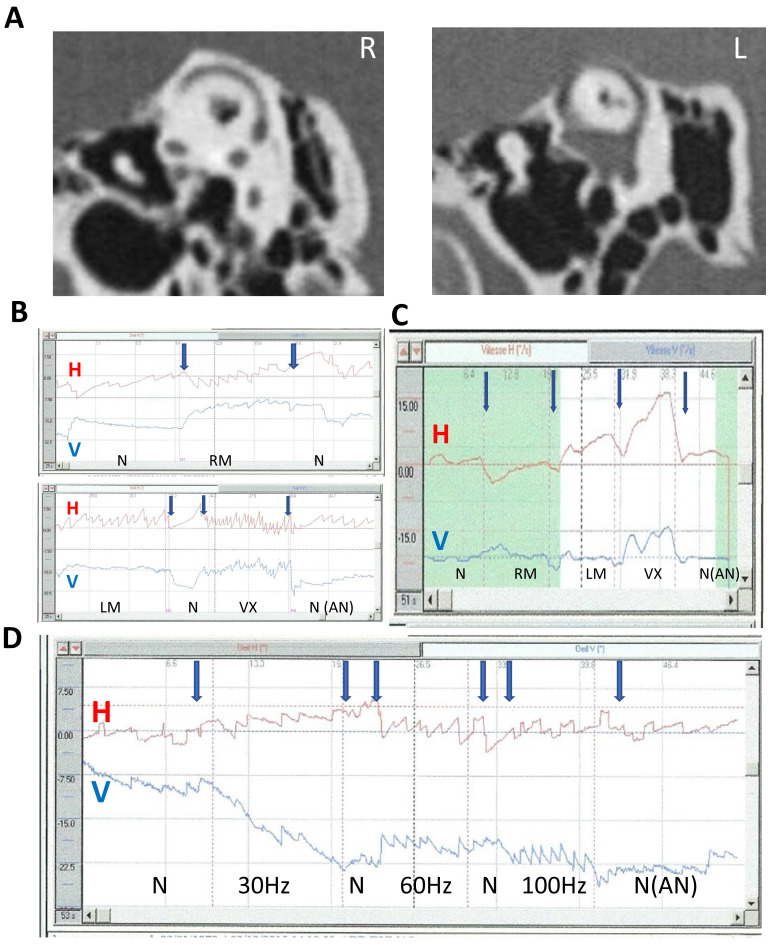
SVIN direction upon stimulus location and frequency. Modified from Dumas et al., 2023 [24]. Example of a left SCD (2D recording; stimulation 100 Hz); (**A**) CT scan of temporal bone reformatted in the Pöschl plane; (**B**,**C**) influence of stimulus location on SVIN direction—(**B**) direct 2D recordings at 100 Hz on LM, RM, VX locations: responses are higher on Vx stimulations. A moderate after-nystagmus (4°/s) is observed after VX stim. The Hor component direction is different based on mastoid location side of stimulation (SVIN beats toward the right on RM location and toward the left on LM location) and is different on RM location vs. VX location). The vertical component observed on vertex location is down-beating. LM: left mastoid stimulation; RM: right mastoid stimulation; Vx: vertex stimulation—(**C**) slow-phase velocity (SPV) 2D recording at 100 Hz: order of stimulation: RM, LM, and then vertex (VX). The stimulation on vertex is ipsilaterally beating to the lesion and shows a higher response than on mastoids. After stimulation withdrawal, an after-nystagmus with a dominant horizontal component; (**D**) influence of stimulus frequency (30 to 100 Hz) on SVIN direction. Stimulation on vertex 2D recording. Change in the horizontal component: at 30 Hz right-beating SVIN; at 60 and 100 Hz left-beating SVIN.

The directions of horizontal and torsional SVIN components changed depending on stimulus location: 50% for RM vs. LM and 55% for Vx vs. mastoids. An example is shown in Figure 5C. In this same patient, the SVIN horizontal component also changed with frequency (30 Hz vs. 60 and 100 Hz) (Figure 5D).

**Figure 6:**
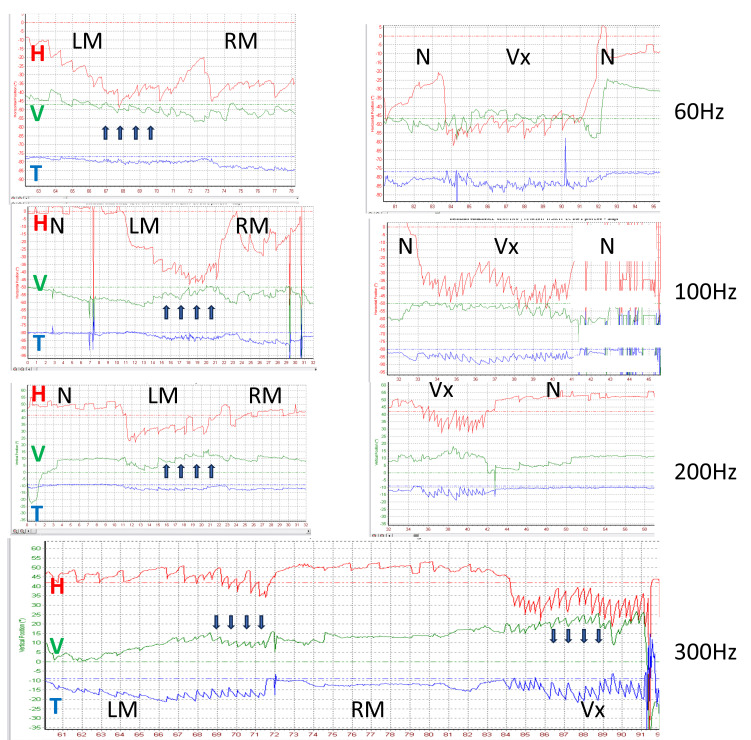
SVIN vertical direction changing following stimulus frequency. Left uSCD. Three-dimensional recordings. Vertex stimulation 60 to 300 Hz SVIN vertical component changing following frequency 60 to 300 Hz. The horizontal component is left beating at all frequencies; higher responses are observed on Vx stimulation. The torsional component is left-beating at all frequencies; higher responses are observed on Vx stimulation. The vertical component is up-beating (black arrows) at 60, 100, and 200 Hz and is down-beating (black arrows) at 300 Hz; higher responses are observed on Vx stimulation LM: left mastoid; RM: right mastoid; Vx: vertex stimulation; N: no stimulation.

The SVIN vertical component changed with changing stimulus frequency in 25% of uSCD patients stimulated at frequencies from 30 to 800 Hz (Figure 6).

**Figure 7:**
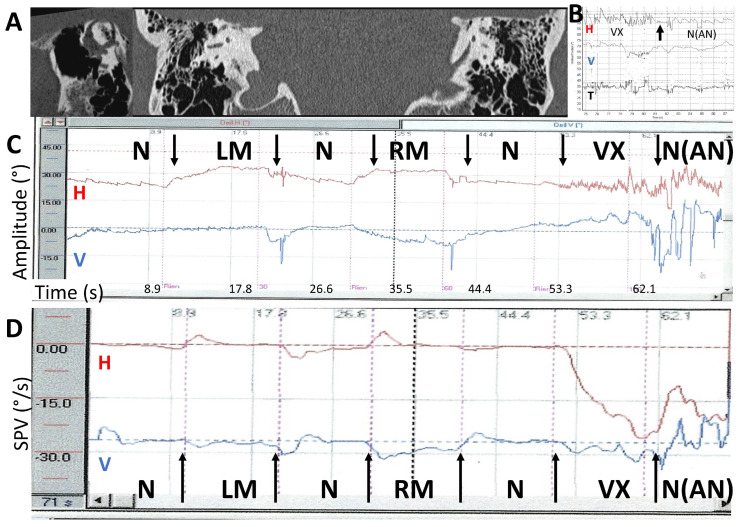
Example of a strong after-nystagmus following a vertex simulation in a right SCD. Two- and three-dimensional recordings (modified from Dumas et al., 2023 [24]). Order of stimulation: RM, LM, and then Vx. Stimulation 100 Hz. (**A**) Temporal bone CT scanner. Frontal slices and images reformatted in the plane of the right superior SCC. The dehiscence is close to the sup SCC ampulla (anterior arm of the sup SCC); (**B**) 3D recording. Vx stimulation response and the after-nystagmus; (**C**) 2D recording: Direct trace: Stimulus 100 Hz. LM: left mastoid stimulation; RM: right mastoid stimulation; VX: vertex stimulation; N: no stimulation. No detectable response after mastoid stimulation. Strong and abrupt response after vertex stimulation followed by an important after-nystagmus mimicking a burst of “spontaneous nystagmus” (SPV = 17°/s). AN: after-nystagmus; (**D**) 2D recording. Slow-phase velocity (SPV). 100 Hz. RM, LM, VX stimulations.

An after-nystagmus at 100 Hz after vertex stimulation in these series was observed in 25% of cases, usually lasting 5–10 s (moderate as in Figure 2), but three other patients showed a strong after-nystagmus (lasting > 20 s) (Figure 7).

In 52% of these cases, the three components (H, V, T) were equivalent and identified (the vertical component in this situation was most often down-beating in 75% and up-beating in 25% of patients with a positive SVIN) at 100 Hz (Figure 6, Table 2).

In 14% of patients with a positive test on vertex stimulation, a primarily vertical down-beating nystagmus was enhanced when the gaze was directed toward the plane of the dehiscent superior SCC, or of the most dehiscent or most symptomatic side (hearing loss, autophony) in the case of asymmetric bSCD.

In this study, a 100 Hz vertex stimulation provoked a reproducible ipsilaterally beating nystagmus in 82% of cases; however, mastoid stimulations showed in 51% of cases a direction-changing SVIN following the stimulated side (Table 1 and Table 2) (Figure 5), unlike the data from uVL patients which, were not direction-changing according to the side stimulated.

The sensitivities (Se) for SVINT, AC Tullio phenomenon (ACTP) with an observed nystagmus, and Hennebert sign were 92%, 35%, and 30%, respectively (Table 1). In the series of patients recorded with a 3D device, Se in bSCD and uSCD was not significantly different. On mastoid location stimulation, the horizontal SVIN SPV obtained in 12 uSCD and 9 bSCD showed a tendency to be smaller in bSCD (3.8°/s ± 1.9) than in uSCD (6.2°/s ± 6.2). Conversely, the vertical component tended to be larger in bSCD (2°/s ± 3.2) than in uSCD (1.7°/s ± 1.5). After vertex location stimulations, the results in these two populations were not significantly different but showed higher amplitudes.

## 4. Discussion

The different clinical data in the most recent publications are in accordance with predictions in recent physiological works [25,26,27,28] SVIN when interpreted in the light of these recent works shows a new insight and confirms its value in the armamentarium of first-line vestibular tests in SCD diagnosis. However, third mobile window syndrome most often described in SCD is not specific to this pathology [29] and SVIN is not specific to SCD diagnosis; it may be observed in other third mobile window syndromes, as reported by White [30]. This author observed a strong (45°/s) ipsilateral beating nystagmus induced by vibration in one of her five patients with enlarged vestibular aqueduct (VAD) syndrome.

Cremer, Minor, and Zee, in their presentation at the XX^th^ Barany Society in 1998, reported “a nystagmus produced by mastoid vibration in patients with a Tullio phenomenon” [16]. They suggested a link between these two phenomena. The first 2D recording of an SVIN obtained at 100 Hz in a patient with unilateral SCD was reported by Dumas et al. as early as 2005 [8]; in this patient, the recorded nystagmus showed a vertical component and an associated horizontal component ipsilaterally beating. Since then, other authors have published observations and recordings of nystagmus provoked by BC in SCD [9,10,17,18,19,20,21,22,23]. These results, variability of nystagmus direction depending on stimulus location and frequency, and an after-nystagmus, not usually observed in uVL patients, are discussed below. According to the literature, the sensitivity range of SVIN in SCD is between 55% [23] and 100% [19].

### 4.1. Optimal Location of Stimulation in SCD

#### 4.1.1. BC Stimulation Locations

Different BC stimulation locations have been described: mastoid [9,18,19], occipital [10], frontal area (Fz), and vertex (Vx or Cz) [9,19,21] regions. The only statistical analysis comparing SVIN SPV for vertex vs. mastoid locations showed that the vertex was more efficient than the mastoid location at 100 and 300 Hz but not at other frequencies [9] (Figure 2). Suboccipital stimulations have also been described and suggested to be very efficient to provoke a nystagmus by White et al. [10] but without statistical data. This last location is otherwise in close proximity with the cervical muscular region.

Vx stimulations are more efficient to reveal an SVIN in SCD patients than in uVL patients [9]: in uVL, mastoid stimulations elicited a contralateral horizontal beating SVIN in 100% of cases, and on Vx stimulations, a positive horizontal SVIN in 75%; conversely, in uSCD, the horizontal component was obtained in 57% of cases on mastoid stimulations and in 92% of cases on Vx stimulations. For the torsional component, in uVL this was 75% on mastoids and 44% on Vx; conversely, in uSCD, it was 50% and 78%, respectively. This result is independent of the order of stimulation location in uVL. The stimulation location in Vx tended to be more efficient when Vx was stimulated after mastoids in uSCD [24].

#### 4.1.2. Possible Explanations for Variable Results in SCD Patients

*Why are vertex stimulations usually more efficient than mastoid stimulations in SCD?*

In patients with SCD, BC stimulation is transmitted to inner ear structures by compression waves through the temporal bone as well as by compression waves through the brain and cerebrospinal fluid (CSF) [25] via dehiscence. Conversely, uVL patients have no middle fossa fistula and show mastoid stimulations that are significantly more efficient than vertex stimulations [9,17]. The bone conduction contribution due to cerebrospinal (CSF) transmission of vibrations under normal conditions is negligible, as mentioned by Stenfeld et al. [31], but may become dominant in cases of middle fossa fistula represented by SCD. In this condition, vertex stimulation may be more efficient than that in uVL patients with an encased labyrinth, where, conversely, SVIN responses on both mastoids are reproducible and beat toward the same direction, corresponding to the stimulation of type I receptor hair cells on the intact side.

The role of soft tissue or CSF has been suggested to contribute to BC by Freeman et al. [32]. A vibration transmitted via the middle cerebral fossa fistula in SCD has already been described by the Cleveland team, in 2007 [10], and suggested by Dumas et al. [9,17].

*Stimulus location modifies responses in SCD after SVINT and BC VEMP.*

In patients with SCD stimulated at the vertex, the VIN direction is most often ipsilateral to the lesion, but may change (in 50% of cases) depending on the side of the stimulated mastoid [13,24]. Vx stimulations are more reliable and are used as referentials in clinical practice to specify the VIN direction.

For bone-conducted (BC) oVEMPs, Manzari et al. [13] demonstrated that stimulations at 500 Hz were more sensitive (87%) than the AC Tullio phenomenon observed in 57% and the Hennebert sign observed in 30% of their 24 cases. Moreover, for oVEMPs, Fz stimulation close to a frontal location induced larger N10 on the contralateral eye than Cz or top-of-cranium stimulations. These authors inferred the change in direction of the wave stimulating the otoliths following the stimulus location and suggested that an Fz stimulus location generated a compressional wave with a rostrocaudal direction, which was more efficient for stimulating the hair bundle at the level of the utricular macula than the Cz location, which created a wave perpendicular to the macula. Thus, similarly for SVIN, the force vector direction generated by the compressional wave may influence the activation of cupula hair cells following the stimulus location.

### 4.2. Stimulus Optimal Frequency—Frequency Spectrum Sensitivity for SVIN in SCD

In uVL patients, the optimal frequency is 100 Hz (no responses observed at 500 Hz) [9]; however, in SCD patients, frequency sensitivity is extended toward higher frequencies and shows more favorable responses at around 400 Hz. Good responses may be observed between 60 and 800 Hz [9]. This extension toward very high frequencies corresponds to the observations in animals by Dlugaiczyk et al. [33] and to the descriptions with oVEMPs (2000–4000 Hz) by Manzari et al. [13]. In some individual patients, the SVIN direction may change depending on the stimulus frequency (e.g., 30 vs. 100 Hz or 100 Hz vs. 300 Hz) (Figure 5 and Figure 6) [24]. These optimal responses observed for SVIN at around 400–500 Hz [9] in patients with SCD correspond to BC facilitation with lower impedance, as described for audiological explorations by Songer et al. [25].

### 4.3. Characteristics of the Nystagmus Obtained in SCD (Direction, Components)

*SVIN 3D recordings in patients with SCD show most often (in 56% of positive cases) three components* [9,17] (Figure 2). This suggests a more global stimulation than the sole superior SCC stimulation. The horizontal component suggests the contribution of either otolith structures (utricle) or horizontal SCC.

White et al. (2007) [10] suggested that only the superior SCC was stimulated by vibrations that induced a VIN with either a torsional (most often ipsilaterally beating) or vertical component (most often down-beating). This has also been suggested by Aw et al. with the vibration-induced vestibulo-ocular reflex (ViVOR) [18] considering the eye slow-phase movement. These authors used scleral search coils and short-duration stimulation at 500 Hz with target fixation, which can explain the negligible horizontal response by inhibition.

A horizontal component associated with a vertical component was recorded with vision denied for the first time by Dumas et al. in 2005 [8] and was later reported by Park et al., 2014 [20], Batuecas et al., 2022 [21], and Koo et al., 2010 [34]. Dumas et al. [9] also observed a torsional ipsilateral beating nystagmus and a nystagmus with a vertical component (down-beating nystagmus in 40% and up-beating in 60% of cases), which they attributed to a probable response of the superior SCC. The variable vertical direction (up- or down-beating nystagmus) and horizontal component could not be explained by a single stimulation of the superior SCC. The vertical nystagmus component direction variability could be explained by the current concept of the Tullio phenomenon related to the flow of the endolymph and nonlinear fluid pumping [26,27,28], which will be detailed below. For the horizontal component, stimulation of structures other than the superior SCC was suggested accordingly to what is known from physiology [35,36].

The implication of the utricle, initially described by Tullio as a co-stimulation [37,38,39,40], may explain the horizontal component of SVIN and was clinically supported by Halmagyi et al. [38] and Dumas et al. [9]. Park also suggested a contribution and co-stimulation of the lateral SCC (2014) [21] to explain the important horizontal slow phase of the ocular movement (ViVOR) component during mastoid stimulations. A possible contribution of the horizontal canal through the spread of regional mobilization of endolymphatic fluid has also been suggested in animals by Carey et al [41]. The implication of an asymmetric concomitant stimulation of otolith structures in SCD patients is otherwise corroborated in the clinical setting by abnormal otolithic test results (ipsilateral hyperexcitability of cVEMPs and oVEMPs) not only with sounds (air-conducted stimulations) but also after bone-conducted vibration stimulations [14,15,38,42].

The SVIN horizontal component frequently observed in SCD cannot be attributed to an unlikely concomitant horizontal SCC pathology. Dumas et al. [9] observed in their series in SCD patients a caloric test seldom modified (3 cases of 23 SCD) and an HVHIT gain asymmetry in 1/22 cases. However, this interaction remains possible in some cases. An anterior SCC VHIT gain asymmetry was more frequently observed in 5/22 patients.

*Why may the VIN vertical direction vary in different patients stimulated with identical stimuli?*

One possible explanation for the SVIN vertical component direction, which differs between patients (up- or down-beating nystagmus), could be that patients with larger dehiscence (>6 mm) may have a superior SCC “auto-plugged” by the overlying cerebromeningeal tissue and thus less stimulated (this corresponds to the frequent superior canal VHIT gain diminution observed in these patients in the literature [43] and shown in Table 2). In this condition, BCV stimulation of the inner ear structures may induce an up-beating nystagmus because the ipsilateral posterior SCC response is less or possibly no longer canceled by the superior SCC response.

*How should we interpret the results in bilateral SCD?*

In bSCD, vertex stimulations usually induce in 75% of positive cases a nystagmus beating toward the more excitable side with the larger hearing loss or audiological symptoms, which usually corresponds to the side of the larger lesion [9,24].

There is otherwise individual patient anatomic variability in SCD, and recent papers [44] showed that 18% had a concomitant additional ipsilateral dehiscence. Some of these dehiscences may have a significant impact on SVIN components.

*To summarize*, SVINT using BC vibration appears to stimulate all vestibular sensory structures. SVIN usually shows three components corresponding to a global and concomitant stimulation of different inner ear structures (torsional and vertical components corresponding probably to superior SCC stimulation and a horizontal component possibly related to the lateral SCC stimulation or to the utricle stimulation) in accordance with physiology [35,36].These structures are otherwise explored in clinical practice using different tests that separately address the superior SCC (e.g., the Hennebert sign, which shows classically a vertical and torsional nystagmus corresponding to a superior SCC response) and otolith structures (particularly utricle), explored through AC or BC oVEMPs.

### 4.4. SVIN as a Bone-Conducted Tullio Phenomenon (BCTP)

The precise mechanism of this phenomenon appears to provide an understanding of the variable results between (and even within) patients and investigators. Here, we consider the apparent discrepancies concerning the direction-changing VIN nystagmus depending upon stimulus location [8,10,13], frequency [9,24], and the presence in a few patients of an after-nystagmus (AN) (persistent VIN after stimulus withdrawal), usually unobserved in uVL lesions [9,17,24].

Dumas et al. [24] link the three main points observed with AC sound in animals with artificially created dehiscence by Tullio in 1929 [5,6] (i.e., imbalance, sway and nystagmus in the plane of the dehiscent canal, and vestibular discomfort) to what is observed after cranial vibrations in clinical practice. The vertex location appears to be particularly effective in inducing nystagmus, associated with reports of dizziness [9,24].

These clinical results cannot be explained by only the third window mechanism associated with bone conduction facilitation toward the lesion side; they need to be interpreted in light of recent data explaining the Tullio phenomenon reported by Iversen et al. (2018) [26] and Rabbitt et al. [27].

*Role of endolymphatic flow created by vibrations.* The mechanism by which BCV activates SCC afferent neurons in patients with SCD appears to be that traveling waves are generated in the endolymph, initiated at the site of dehiscence [24], and travel from that site in both directions around the canal. Recent direct physical measurements of fluid flow in an artificial dehiscent SCC demonstrate this mechanism [26]. This mechanism is confirmed by physiological evidence from recording single SCC neurons in animals after artificial SCD. These data demonstrate that vibration causes two modes of activation of SCC neurons after SCD:

(1) Cycle-by-cycle phase-locked activation of action potentials in SCC afferents with irregular resting discharge;
(2) Cupula deflection by fluid streaming caused by the traveling waves of fluid displacement initiated by sound or vibration at the point of dehiscence. This fluid flow causes a slow deflection of the cupula, allowing for a slow return at the end of BCV stimulation and thus accounting for after-nystagmus. This cupula deflection stimulates neurons with regular resting discharge that are not directly activated by cycle-by-cycle phase-locked vibration [24,26]. The direction of the fluid current depends on the location of the stimulus, the location and size of the dehiscence, and the frequency of the stimulus [26,27,28]. The direct measures showed that the direction of fluid flow changed with frequency.

These two mechanisms explain the following:

(1) Direct stimulation on the affected side of the type I vestibular receptor hair cells (and afferent neurons with irregular neural discharge) at high frequency favored by BC facilitation toward the side of the dehiscence in relation to the third window mechanism [25]. This explains the most commonly observed ipsilateral excitatory nystagmus.
(2) The second mechanism [26,27] explains, in some SCD patients, a prolonged VIN after stimulus offset (after-nystagmus) mimicking, in some cases, an after-nystagmus after the end of an angular acceleration stimulus.

As shown by Curthoys et al., these two co-existing mechanisms—cycle-by-cycle activation and fluid flow—work together or they can oppose each other. After SCD, if acoustic streaming deflects the cupula in an inhibitory direction, all the receptor hair cells and afferents from that canal will be progressively silenced. Consequently, maintained sound or vibration stimulation silences the cycle-by-cycle phase-locked activation of irregular afferents. Thus, these two mechanisms may in some conditions be complementary and in others in competition or antagonistic, explaining the possible variability in the resulting SVIN direction.

This corresponds to the frequent horizontal direction-changing nystagmus observed on mastoid stimulation depending on the side of the stimulated mastoid (Figure 3 and Figure 4).

It also explains the variability of the vertical component in the literature (up- or down-beating nystagmus) depending on stimulus location [9,10,17,24] and sometimes on frequency [9,24] (Table 1, Figure 6).

*Why does BCV at Vx in patients with SCD induce an ipsilateral SVIN (observed in 80% of cases)?*

In their animal model, Iversen et al. [26] showed that the direction of the fluid flow caused by BCV stimulation after an artificial SCD depended on the size of the dehiscence, the location of the dehiscence, and the frequency of the stimulus. The frequencies from 100 to 500 Hz caused excitation and frequencies >500 Hz (up to 800 Hz) caused inhibition and then again excitation at even higher frequencies in their toadfish model. The reversal appears to depend on which of the two traveling waves causes cupula deflection. They also recorded single SCC neurons, which confirmed the predictions from their measurements and modeling. It also explains the nystagmus observed in humans.

*Such stimulation by vibration usually induces concomitant dizziness or unsteadiness*, possibly more suggestive of otolith symptoms than canal symptoms. Unsteadiness or discomfort often associated with nausea has been reported by Dumas et al. [9] in 16/27 SCD patients (60%) repeatedly stimulated. This has already been described with air-conducted sound (ACTP) by Minor et al. [1] and Ward et al. [4].

### 4.5. Sensitivity of SVIN to Detect SCD Compared with Other Bedside Explorations or Vestibular Test Explorations

Classical bedside examinations in SCD are compressional tests for nystagmus induced by AC sounds (Tullio phenomenon) or by the Valsalva test with a pinched nose or the Hennebert sign [2,4]. These tests provoke a positive pressure in the middle ear that induces, via the oval window, an ampullofugal endolymph flow and nystagmus for the anterior SCC. The sensitivity varies for the AC Tullio phenomenon between 25% and 80% and for the Hennebert sign between 21% and 25% (Table 1).

Mehta et al. observed positive Hennebert sign, ACTP, and a positive SVIN in 20%, 30%, and 40% of cases, respectively, in SCD [23]. A positive SVIN was reported by Batuecas et al. [21] in 62% of their cases and in 82% and 87% of cases by Dumas et al. [9,17]. These results suggest a possible higher sensitivity and efficiency of the BC Tullio phenomenon (BCTP) represented by SVINT over the AC Tullio phenomenon (ACTP).

Similarly, BC stimulations for oVEMPs are more efficient than BC cVEMPs in uncovering SCD patients but are not more efficient than AC oVEMPs when compared with air-conducted stimulations [11,12].

Otolith tests (oVEMP, cVEMP) are currently described as the most efficient in SCD diagnosis; they are positive in 80–90% of cases with ACS stimulations [11,14] but also after BC stimulations [42].

The value of SVINT, a recent exploration that is less specific but more sensitive, rapid, and is used as a non-intrusive bedside test, is to show (or suggest) an SCD and symptoms related to a third window mechanism. This test is interesting to report because it is in SCD more often observed than other classical objective tests such as the Hennebert sign and air-conducted Tullio phenomenon.

SVIN extension of sensitivity toward very high frequencies is similar to what is observed with BCV for oVEMPs and cVEMPs, which after CT-verified SCD, show an extension of sensitivity toward very high frequencies (4000 Hz). This was particularly ob served by Manzari with oVEMPs in 100% of his 22 patients with SCD [13].

In summary, VEMPs appear to offer a non-invasive test providing quantitative data and are the most useful test for surgical decision-making. SVIN, considered as a BCTP, has recently showed its useful contribution in SCD diagnosis among other bedside examination tests such as the Hennebert test and complements the AC Tullio phenomenon with possibly more sensitivity.

### 4.6. Limitations

A limitation is the small series reported (6 to 40 patients). SCD is an infrequent pathology (0.5% in the series of 1000 temporal bone archives of Carey et al. [45]) often misrecognized in clinical practice [46,47,48].

The results show strong heterogeneity in the SVIN protocol between series considering that different authors use different stimulus topographies and frequencies.

Vibrators using adequate frequencies from 100 to 800 Hz may miss the nystagmus mainly when the vibrator amplitude is too small to provoke a jerk effect on the inner ear hair cell bundles, which work as seismic receptors, as already described by I.S. Curthoys. Dlugaiczyk et al. [33] showed that the vibration needs accelerations of the head between 0.1 and 0.4 g to elicit a neural response, which are values below 2 g, non-invasive in humans. Otherwise, it has been shown that for a constant frequency, vibrators delivering amplitudes of vibrations lower than 0.1 mm are inefficient to reveal a nystagmus. The series using frequencies only around 100 Hz may miss the nystagmus, which is sometimes visible at 400 Hz or at higher-frequency vibrations. The SVIN is sometimes very poor with small SPV at 100 Hz [9].

Using eye fixation minimizes or suppresses by inhibition the horizontal component. Thus, it is mandatory when using SVINT to mask the non-recorded eye to improve the response and suppress this bias [24].

## 5. Conclusions

This review offers insights into SVIN as a recent and efficient tool among other vestibular tests to investigate SCD. SVIN is not specific for SCD but may be observed in other third mobile window syndromes. The main leads of interpretation must consider this test as a BC Tullio phenomenon. The Tullio phenomenon initially described after AC stimulation is currently most often observed in SCD patients but is not specific to this pathology. The symptoms of dizziness and nystagmus provoked by BC vibrations in SCD show that SVIN constitutes a more efficient alternative to the air-conducted Tullio phenomenon to reveal the pathology. SVIN in SCD constitutes the two targets initially described by Tullio after air-conducted sound stimulation by the dehiscent SCC and utricle. SVIN expresses these contributions as vertical and torsional components for the superior SCC response and as a horizontal component for the probably associated utricle and/or lateral SCC contribution. Interpreting SVIN results in patients with SCD as a BC Tullio phenomenon is consistent with SVIN direction depending on stimulus location and stimulus frequency and, in a few patients, with an after-nystagmus related to the flow created by the pumping mechanism and stimulating receptor hair cells of the cupula. The flow phenomena are nonlinear and the instability explains the variability observed in some patients and discrepancies between investigators. The resulting SVIN direction depends on the complementarity or antagonism of two contributing mechanisms: (1) cycle-by-cycle phase-locked activation of SCC afferents with irregular resting discharge, and (2) cupula deflection induced by fluid streaming caused by the traveling waves. Skull-vibration-induced nystagmus in SCD patients shows, on vertex stimulations, an excitatory SVIN with torsional and horizontal components most often ipsilaterally beating toward the lesion. Bone-conducted stimulations in SCD using the skull-vibration-induced nystagmus test should be systematically included in the armamentarium of third window syndrome screening tests.

We recommend that future research should promote non-invasive devices for stimuli, use protocols with vision denied, and conduct a systematic study of very high frequencies up to 800 Hz. A sufficient duration of stimulation is otherwise advised. 

## Figures and Tables

**Table 1:** Literature data on skull-vibration-induced nystagmus (SVIN) in superior semicircular canal dehiscence. Modified from Dumas et al., 2023 [24].

	Se (%)	Cohort Size	Patient Count uSCD/ bSCD	Stimulus Locations Optimal Location	Stimulus Frequency (Hz)	SHC Num *(%)*	SVC Num *(%)*	STC Num *(%)*	SVIN DCF Location Num - *(%)*	SVIN DCF Frequency Num - *(%)*	After-nyst Num	AC Tullio Num - *(%)* Hennebert Num - *(%)*
**White et al., 2007 [10]**	100	8	6	RM, LM, Vx, SO	100	ND	Down 2 Up 1	Contr3 Ipsi 2	1/8 *(12)*	ND	1/8 combined with positional nyst.	ND 2/8 *(25)*
			2	Opt. SO		ND	Down 1 Up 0	2				
**Schmerber et al., 2008 [22]**	-	6	4	RM, LM Vx	100	Ipsi 3 *(50)*	Down 2 Up 2	ND	ND	ND	ND	rarely
			2	Opt Vx	-	0	0	ND				
**Manzari et al., 2008 [19]**	100	16	9 7	RM, LM -	100 -	Poor Poor	Down 7 Up 2 Down 4 Up 1	Ipsi 4 Contr 0	HC: 7/9 VC: 7/9 TC: 6/7	ND	ND	9/16 *(56)* ND
**Aw et al., 2011 [18]** **(ViVOR)**	100	17	12	RM, LM	500	Small (negligible)	ViVOR up	ViVOR contr	0 TC: RM/LM	ND		9/12 *(80)* ND 5/5 *(100)* ND
			5	-	-	Small	ViVORup++					
**Dumas et al., 2014 [17]**	82	17	17	RM, LM Vx Opt Vx	100	Ipsi *(70)*	Up *(47)*	ipsi *(62)*	HC 2/17 VC 1/17 TC 2/17	ND	4/17	4/17 *(24)* ND ND
			0	0		0	0	0				
**Park et al., 2014 [20]** **(ViVOR)**	90	10	9	RM, LM	100	ViVOR Ipsi *(18)* Contr *(70)*	Down *(21)* Up *(75)*	1	ND	ND	ND	8/10 *(80)* ND
			1	-	-	-	-	-				
**Mehta et al., 2015 [23]**	55	38 CD (50 CD+T)	ND	RM, LM	ND	ND	ND	ND	ND	ND	ND	10/38 *(26)* 8/38 *(21)*
**Dumas et al., 2019 [9]**	86 54	40	27	RM, LM, Vx	100–800	Ipsi *(85)* Contr *(2)*	Down *(40)* Up *(60)*	Ipsi *(57)* Contr *(2)*	2/10 *(20)*	2/8 *(25)*	1	5/20 *(25)* ND ND ND
			13	Opt Vx			Down *(25)* Up *(75)*					
**Batuecas et al., 2022 [21]**	66	30	25	RM, LM Vx	100	6/30	Up 14/30	ND	ND	ND	ND	ND ND ND ND
			5	Opt: ND		4/9	Up 4/9	ND				
**Dumas et al., 2023 [24]**	91	52	39	RM, LM Vx	30–800	Ipsi 29/36 *(81)* Contr 5/36 *(19)*	Down13/25 *(52)* Up 12/25 *(48)*		21/37 *(56)*	2/8 *(25)*	10/28 *(25)*	12/34 *(35*) 10/35 *(30)*
			13	Opt *Vx = M*								

Se: sensitivity of the test. uSCD: unilateral superior canal dehiscence; bSCD: bilateral superior SCC dehiscence. Nystagmus direction: ipsi: SVIN beating ipsilaterally; contr: SVIN beating contralaterally. SVIN direction for the torsional component is given as follows: an ipsilateral beating nystagmus corresponds to the fast mobilization (jerk) of the eye upper part directed toward the patient lesion. A contralateral beating nystagmus corresponds to the fast mobilization (jerk) of the eye upper part directed away from the patient lesion (or toward the intact side). ViVOR: vibration-induced vestibulo-ocular reflex; the directions mentioned indicate the direction of the eye slow-phase displacement. Stimulus location: RM: right mastoid; LM: left mastoid; Vx: vertex, SO: suboccipital. Opt: optimal location. DCF: direction changing following. SHC = SVIN horizontal component direction; SVC = SVIN vertical component direction; STC = SVIN torsional component direction. HC: horizontal component; TC: torsional component; VC: vertical component. AN: after stimulus nystagmus. ND: no data.

**Table 2:** SVIN sensitivity in 39 uSCD. Characteristics of the VIN components (modified from Dumas et al. [24]).

Series	Nb	Age (Mean +/− SD)	Gender M/F	Size Mean +/− SD	SVIN Se VX + M Nb, *(%)*	Optimal Location Nb, *(%)*			SVIN Direction On VX Stim. Nb, *(%)*		SVIN CDFL Vx/M LM/RM	SVIN CDFF 60–800 Hz	After-nyst.	oVEMP	cVEMP	VHIT Ipsi Hypo Nb, *(%)* Gain Mean+/− SD	ACTP (Tullio)	Hennebert
						Vx	Vx = M	M	H Comp	V Comp								
**GD**	12	62.2 10.32	M = 5 F = 7	4.55 1.80	11 *(91)*	9 *(82)*	2 *(18)*	0 *(0)*	Ip:10/11 (91) Ctl:1/11 *(9)*	Up: 2/8 Down: 6/8	6/11 *(54)*	2/8 *(25)*	3/12 *(25)*	8/9 *(88)*	9/10 *(90)*	2/12 Gain ND	4/10 *(40)*	2/10 *(20)*
**AC**	27	64.03 9.80	M = 15 F = 12	3.20 1.29	25 *(93)*	2 *(8)*	9 *(35)*	15 *(57)*	Ip:19/25 *(75)* Ctl:4/25 *(15)*	Up: 10/17 Down: 7/17	15/26 *(57)*	ND	7/26 *(26)*	23/27 *(85)*	24/27 *(96)*	21/27 Gain 0.56 0.17	8/24 *(33)*	8/23 *(35)*
**Total**	39	63.4 9.86	M = 20 F = 19	3.58 1.55	36 *(92)*	11 *(30)*	11 *(30)*	15 *(40)*	Ip:29/36 *(81)* Ctl:5/36 *(19)*	Up: 12/25 Down: 13/25	21/37 *(56)*	*(25)*	10/28 *(25)*	31/36 *(86)*	33/37 *(89)*	23/39 *(58)*	12/34 *(35)*	10/35 *(30)*

GD. (G. Dumas protocol) = Order 1 in 12 patients: Mastoid stimulations before vertex (Vx) stimulation. AC. (A. Castellucci protocol) = Order 2 in 27 patients: Vertex stimulation before mastoid stimulation. M: stimulation on mastoids; Vx: vertex stimulation; LM: left mastoid; RM: right mastoid; Up: up-beating nystagmus; Down: down-beating nystagmus; Stim: stimulation; nyst: nystagmus; numb: number of cases; (%) Gain: gain in % for the superior canal VOR using the VHIT test; Mean: mean value; SD: standard deviation; SVIN CDFL: SVIN changing direction following location of the stimulus; SVIN CDFF: SVIN changing direction following stimulus frequency; ACTP: air-conducted Tullio phenomenon; Hor comp: horizontal component; Vert comp: vertical component; Ipsi: nystagmus beating ipsilaterally to the lesion; Contra: nystagmus beating contralaterally to the lesion.

## Data Availability

This is a review paper with analytic methods (Prisma guidelines). Data are available upon request to the main authors or corresponding author (MD).

## Abbreviations

ACTP	air-conducted Tullio phenomenon
BCTP	bone-conducted Tullio phenomenon
SCD	superior (semi-circular) canal dehiscence
uSCD	unilateral SCD
bSCD	bilateral SCD
uVL	unilateral vestibular loss
BC or BCV	bone-conducted vibrations
SVIN	skull-vibration-induced nystagmus
SVINT	SVIN test
SPV	slow-phase velocity
ViVOR	vibration-induced vestibulo-ocular reflex
